# Integrin α2 in the microenvironment and the tumor compartment of digestive (gastrointestinal) cancers: emerging regulators and therapeutic opportunities

**DOI:** 10.3389/fonc.2024.1439709

**Published:** 2024-11-06

**Authors:** Tiantian Liu, Yanmei Gu, Yuyu Zhang, Yumin Li

**Affiliations:** Lanzhou University Second Hospital, the Second Clinical Medical College of Lanzhou University, Lanzhou, Gansu, China

**Keywords:** integrin α2, digestive cancers, chemoresistance, genomic instability, tumor microenvironment

## Abstract

Integrins are a family of cell surface membrane receptors and play a crucial role in facilitating bidirectional cell signaling. Integrin α2 (ITGA2) is expressed across a range of cell types, including epithelial cells, platelets, megakaryocytes, and fibroblasts, where it functions as a surface marker and it is implicated in the cell movements. The most recent findings have indicated that ITAG2 has the potential to function as a novel regulatory factor in cancer, responsible for driving tumorigenesis, inducing chemoresistance, regulating genomic instability and remodeling tumor microenvironment. Hence, we primarily focus on elucidating the biological function and mechanism of ITGA2 within the digestive tumor microenvironment, while highlighting its prospective utilization as a therapeutic target for cancer therapy.

## 1 Introduction

**Figure 1:**
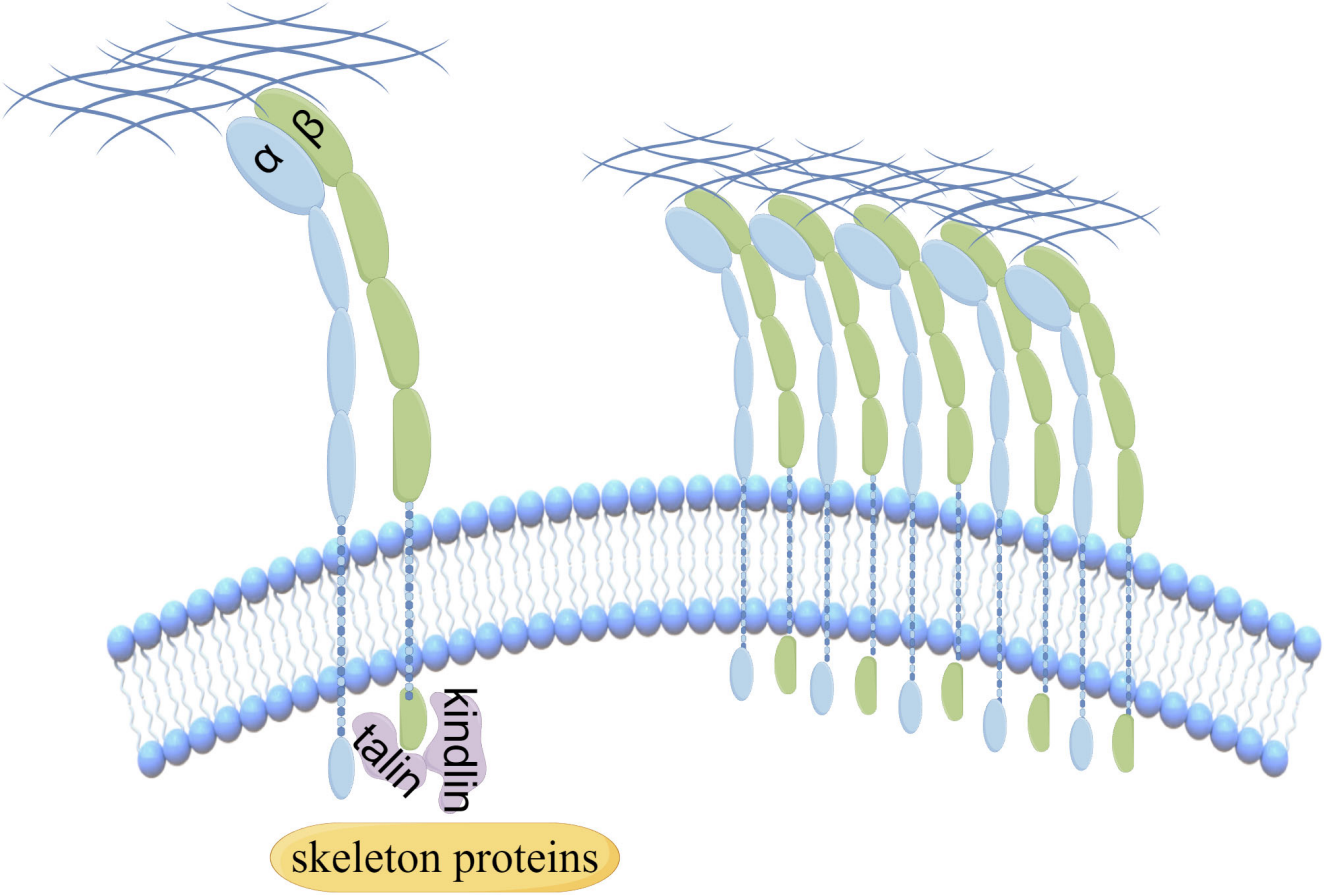
The aggregation of integrins is regulated by the interaction of intracellular regulatory proteins.

**Figure 2:**
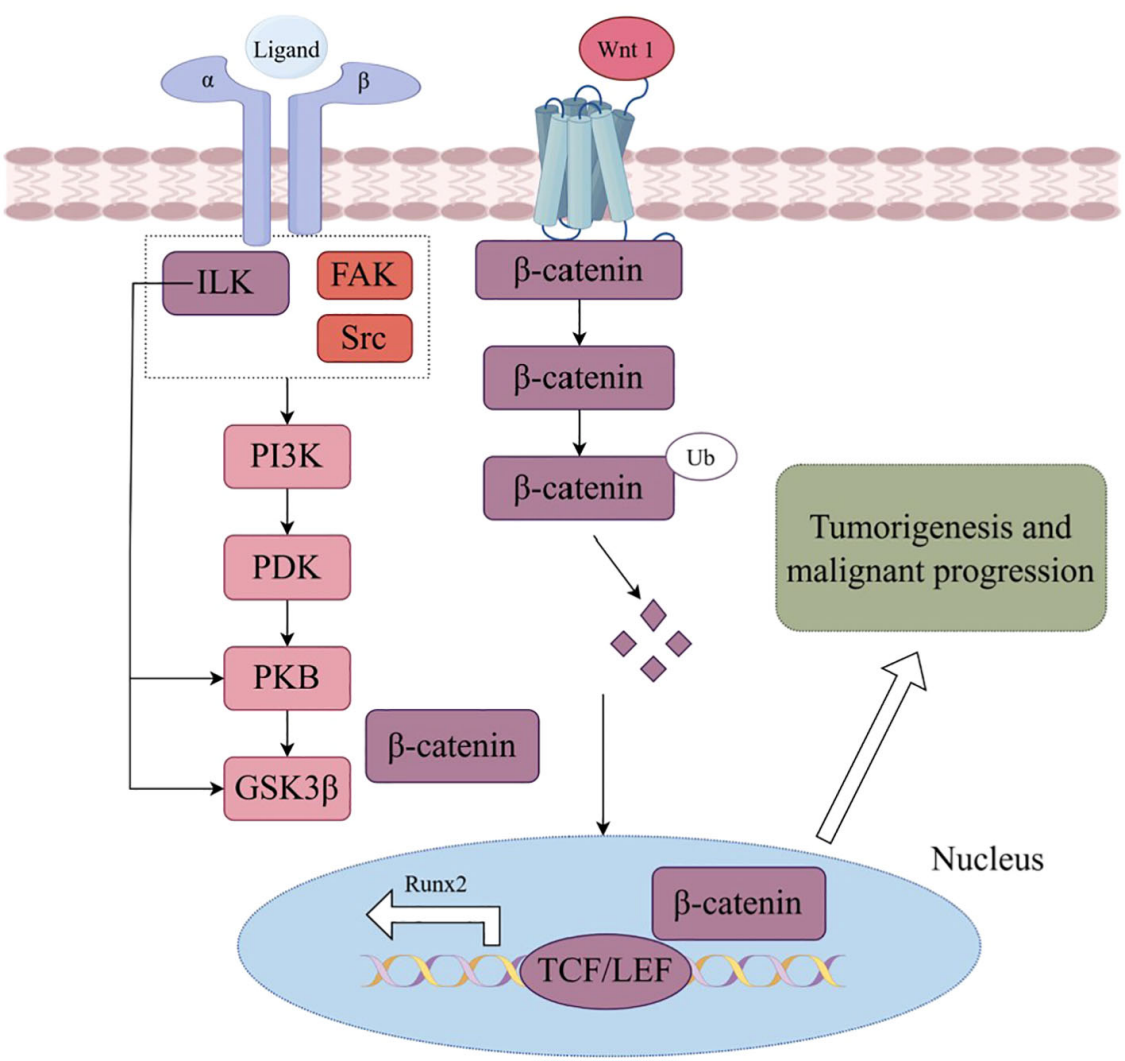
Wnt signaling in integrin triggers phosphatidylinositol 3 kinase (PI3K) activation, subsequently regulating glycogen synthase kinase 3β (GSK3β), ubiquitin (Ub)induced β-catenin degradation, nuclear location and Wnt targeting gene expression, which leading to tumorigenesis and malignant progression. (By Figdraw.).

Integrins, comprising 18 α-integrin subunits and 8 β-integrin subunits, constitute a group of membrane receptors that hold significant importance in diverse physiological processes, encompassing cell motility, skeletal remodeling, and cell growth (1). The α subunit governs the specificity of the extracellular ligand, whereas the β subunit primarily mediates intracellular signaling. Integrin-mediated signaling exhibits bidirectionality, wherein it establishes complexes with extracellular matrix ligands, thereby initiating signals from the external environment towards the interior of the cell (2). Conversely, the initiation of external signals usually begins with the interaction of intracellular regulatory proteins talin and kindlin with the β subunit, subsequently causing conformational changes and ultimately leading to the aggregation of integrins (**Figure 1**). Activated integrin molecules form clusters on the cell membrane, and intracellular segments recruit intracellular signaling molecules and skeleton proteins to provide traction for cell movement and migration. Effective treatments have successfully targeted integrins for cardiovascular diseases, inflammatory bowel disease/multiple sclerosis and dry eye disease. However, clinical development of others has faced significant challenges in the fields of cancer (3). Nowadays, there are studies indicating that the combination of integrin and Wnt signaling could induce tumorigenesis and malignant progression in various cancers (**Figure 2**).

Integrin α2 (ITGA2) typically associates with integrin β1 (ITGB1) to form a heterodimer, serving as a distinctive receptor for type I and Type IV collagen (4). Initially identified on epithelial cells, platelets, megakaryocytes, fibroblasts, and activated T cells, ITGA2 was found to be expressed in these cell types (5). Recently, an increasing number of studies have observed abnormal expression of ITGA2 in various malignancies, exerting a significant influence on multiple facets of tumor initiation and progression, encompassing pancreatic cancer, gastric cancer, liver cancer, breast cancer, and glioma (6–9) as well as it has been demonstrated that loss of ITGA2 leads to reduced proliferation, invasion, and adhesion of glioblastoma cells, which suggested that ITGA2 emerged as a potential therapeutic target in glioblastoma (10). Consequently, attention has been directed towards the role of ITGA2 in tumorigenesis. The emergence of the tumor microenvironment (TME) concept posits that the interaction between tumors and diverse complex components enables tumor cells to evade host immune surveillance (11). This recognition has prompted a paradigm shift in cancer research, redirecting focus from solely investigating tumor cells to scrutinizing the intricate ecosystem encompassing the tumor. Currently, a substantial body of evidence supports the expression of ITGA2 in various constituents of the TME and its participation in the remodeling of said microenvironment (12, 13). Here, our primary objective is to review the biological function and molecular mechanism of ITGA2 in gastrointestinal tumors, alongside conducting a comprehensive analysis of the existing understanding of ITGA2 in the regulation of the TME.

## 2 ITGA2 roles in gastric cancer

According to Lauren’s classification, gastric cancer (GC) can be classified into two distinct types: intestinal and diffuse. Min et al. discovered a correlation between the accumulation of ITGA2 and the occurrence of intestinal precancerous lesions in GC and enhance the migratory ability of GC cells *in vitro (* 14). The promotion of tumor cell migration is an essential requirement for the phenomenon of cancer metastasis, the infiltration of cancerous cells through the basement membrane and subsequent entry into the circulatory system. Among patients with advanced GC, peritoneal metastasis represents the prevailing manifestation, which is associated with a dismal prognosis (15). Zang et al. have successfully identified LPPR4 as a noteworthy biomarker for peritoneal metastasis in GC. Their research has revealed that this biomarker plays a pivotal role in regulating the migration, invasion, and adhesion of GC cells via the Sp1/integrin/FAK signaling pathway (16). Furthermore, in a mouse model of GC with peritoneal metastasis, the inhibition of NF-κB has been observed to effectively hinder the adhesion of cancer cells to the peritoneum by specifically targeting ITGA2 (17). Additionally, the induction of Cyr61 has been found to upregulate ITGA2 through the AP-1 signaling pathway, thereby intensifying the peritoneal metastasis of GC cells (18). The aforementioned findings indicate that ITAG2 plays a crucial role in the peritoneal metastasis of GC. Integrin endocytosis is recognized as a significant mechanism that governs the migration and movement of cancer cells. Integrins regulate cell migration by either preserving the existing cell matrix or forming a new cell matrix via endocytosis (19). Consequently, it is plausible that ITGA2 is implicated in the metastasis of GC through the process of endocytosis.

Chemotherapy resistance is a contributing factor to the unfavorable prognosis observed in patients with GC, leading to both local recurrence and distant metastasis. It has been reported that elevated expression of ITGA2 is evident in chemotherapy-resistant cells. Additional investigations have demonstrated that ITGA2 facilitates chemoresistance in GC by activating the MAPK signaling pathway (20). The investigation conducted by Dong et al. revealed a novel signaling pathway, HMGA2-FOXL2-ITGA2, which has been demonstrated to exert an influence on both chemotherapy resistance and metastasis. Through the utilization of tissue chip analysis, it was observed that the expressions of HMGA2, FOXL2, and ITGA2 were elevated, consequently leading to an augmented risk of metastasis in individuals diagnosed with GC (21). Tumor stem cells are a key regulator in tumor heterogeneity and exhibit a strong association with both cancer progression and drug resistance. Furthermore, Liu et al. have substantiated the resistance of ITGA2 to chemotherapy in GC stem cells (22). These findings imply that the augmentation of chemotherapy sensitivity in GC can be achieved by targeting ITGA2, although the precise mechanism underlying this phenomenon remains elusive. Given the involvement of ITGA2 in the tumorigenesis, metastasis, and drug resistance of GC, it emerges as a promising candidate for the diagnosis and treatment of GC. (Mechanisms and outcomes of ITGA2 in chemotherapy resistance of GC are shown in **Table 1**).

**Table 1:** Mechanisms and outcomes of ITGA2 in chemotherapy resistance of GC.

Molecular types	Mechanisms	Outcomes	References
GC cells	Elevated expression of ITGA2 activates the MAPK signaling pathway	Contributing to chemotherapy-resistant of GC cells	(23, 24)
GC cells	Expressions of ITGA2 are elevated in HMGA2-FOXL2-ITGA2 signaling pathway	Leading to an augmented risk of metastasis in individuals diagnosed with GC	(23, 24)
GC cells	Resistance of ITGA2 to chemotherapy in GC stem cells	Exhibiting a strong association with both cancer progression and drug resistance	(23, 24)

## 3 ITGA2 roles in pancreatic cancer

Pancreatic cancer is a prominent contributor to cancer-related mortality on a global scale (25). ITGA2 has been observed in both normal pancreatic ductal epithelium and in pancreatic ductal adenocarcinoma (PDAC). However, the role of ITGA2 in PDAC remains a subject of debate. In PDAC, the expression of integrin α2β1 is detected in well-differentiated tumors, whereas ITGA2 tend to undergo epitope loss or alter subcellular localization during the cancer progresses. For this, the study concluded that ITGA2 may not be conducive to the malignant progression of PDAC, and this effect may be attributed to the regulation of KLK5 expression (26). Furthermore, it has been documented that ITGA2 exhibits enrichment in pancreatic progenitors, thereby facilitating pancreatic development and regeneration (27).

Fibroconnective tissue hyperplasia is a prominent characteristic of PDAC. Pancreatic stellate cells (PSCs) play a crucial role as fibrotic cells within the pancreas, with collagen type I serving as the primary adhesion molecule in PDAC, induced by PSCs. The secretion of type I collagen by PSCs significantly facilitates cell migration via the integrin α2β1-FAK signaling pathway (28). The upregulation of ITGA2 and ITGB1 heterodimer regulates the degradation of the E-cadherin complex and the translocation of β-catenin to the nucleus, thereby enhancing the migration of PDAC cells (29). Consequently, ITGA2 plays a pivotal role in promoting PDAC metastasis by interacting with type I collagen.

The presence of genomic instability disorder is a notable feature of cancer, and compelling evidence indicates a correlation between ITGA2 and genomic instability in PDAC. Furthermore, recent research substantiates the involvement of ITGA2 in the DNA damage response (DDR), thereby establishing its potential utility as a predictive marker for radiation response. Double-strand DNA breaks (DSBs) represent a main type of DNA damage, and non-homologous end joining (NHEJ) serves as a principal mechanism for repairing DSBs. DNA-dependent protein kinase catalytic subunits (DNA-PKCs) are protein kinases that contribute to the DDR and the preservation of genome integrity. The Ku70/80 heterodimer recognizes the site of DNA damage and interacts with DNA-PKCs to initiate the NHEJ pathway. By competitively binding with the Ku70/80 heterodimer, ITGA2 can impede the activity of DNA-PKCs and the activation of the NHEJ pathway, thereby augmenting the susceptibility of PDAC cells to radiotherapy (30). ITGA2 functions in the regulation of DDR in PDAC, as well as in the modulation of DNA methylation, a prominent epigenetic modification that influences the expression of tumor-associated markers. A comprehensive analysis of PDAC utilizing a DNA methylation assay revealed that reduced ITGA2 methylation and heightened transcriptional activity were correlated with unfavorable survival outcomes in PDAC patients (31). Notably, DNA methyltransferase 1 (DNMT1) serves as an essential enzyme for DNA methylation. In the context of PDAC, ITGA2 disrupts the interaction between DNMT1 and Kindlin2, thereby triggering the degradation of DNMT1 (32). Therefore, the regulatory function of ITGA2 in DNMT1 implies its potential impact on the genetic alteration of PDAC. While the involvement of ITGA2 in PDAC remains a topic of debate, a growing body of research provides evidence supporting ITAG2’s role as an oncogene in the initiation and malignant advancement of PDAC.

## 4 ITGA2 roles in hepatocellular cancer

The biological behaviors of cells, such as cytoskeletal recombination, cell motility, and growth, are primarily governed by the adhesion of cells to the extracellular matrix (ECM) (33). Collagen, being the primary constituent of the ECM, plays a crucial role in preserving structural integrity and significantly regulating diverse cellular functions. An increasing body of evidence suggests that the interaction between ITGA2 and collagen facilitates the migration and metastasis of tumor cells. The upregulation of type IV collagen synthesis and deposition serves as a prevalent biomarker for the advancement of hepatocellular cancer (HCC). Through the integration of clinical data and fundamental experimental assays, ITGA2 engages with type IV collagen to initiate focal adhesion signaling, thereby facilitating the liver metastasis (9). Additionally, an independent investigation corroborated the role of ITGA2 as a diagnostic marker for liver metastasis in colorectal cancer (34). The attenuation of migratory and invasive capabilities of HCC cells within the microenvironment can be achieved by negatively inhibiting the interaction between ITGA2 and type I collagen (35).

The liver is commonly implicated as a site for the metastasis of tumors originated from the digestive system. ITGA2 not only exerts a substantial influence on this metastatic process, but it also exhibits a close correlation with the origin of HCC. The origin of HCC is intricately linked to the dynamic selection of the TME. Through a comprehensive analysis of single-cell transcriptomes in HCC patients, it has been observed that tumor-associated fibroblasts exert a dominant influence in HCC, resulting in a notable level of heterogeneity among tumor cells. This key driving force is mediated by the interaction between type I collagen and ITGA2 (36). Taken together, targeting the ITGA2-mediated interaction between cancer cells and collagen emerges as a promising therapeutic approach for mitigating the risk of cancer metastasis and dissemination.

## 5 ITGA2 roles in colorectal cancer

Current evidence substantiates the role of ITGA2 as a tumor marker for colorectal cancer (CRC), contributing to its malignant biological phenotype (23, 24). The ECM remodeling within the TME is a significant characteristic of malignancy, impacting the localization and migration of cancer cells. The up-regulation of ITGA2 facilitates the migration and proliferation of cancer cells by augmenting the adhesion of CRC cells to collagen and laminin (37). Moreover, the secretion of TGF-α by CRC cells enhances the expression of ITGA2, leading to increased cell adhesion to type IV collagen and promoting the metastasis and dissemination of cancer cells (38). The effect of ITGA2 on the migratory and adhesive capabilities of CRC cells is associated with the activation of the adhesion kinase signaling pathway (39). Anoikis resistance, a critical step during the cancer metastasis, enables cells to survive in the absence of ECM. Studies have indicated that cancer stem cells (CSCs) display heightened expression of ITGA2 and ITGB1, which interact with EGFR to initiate the ERK/Akt-mediated survival pathway, thereby facilitating CSCs in evading anoikis (40).

## 6 The regulation of ITGA2 in tumor microenvironment

**Figure 3:**
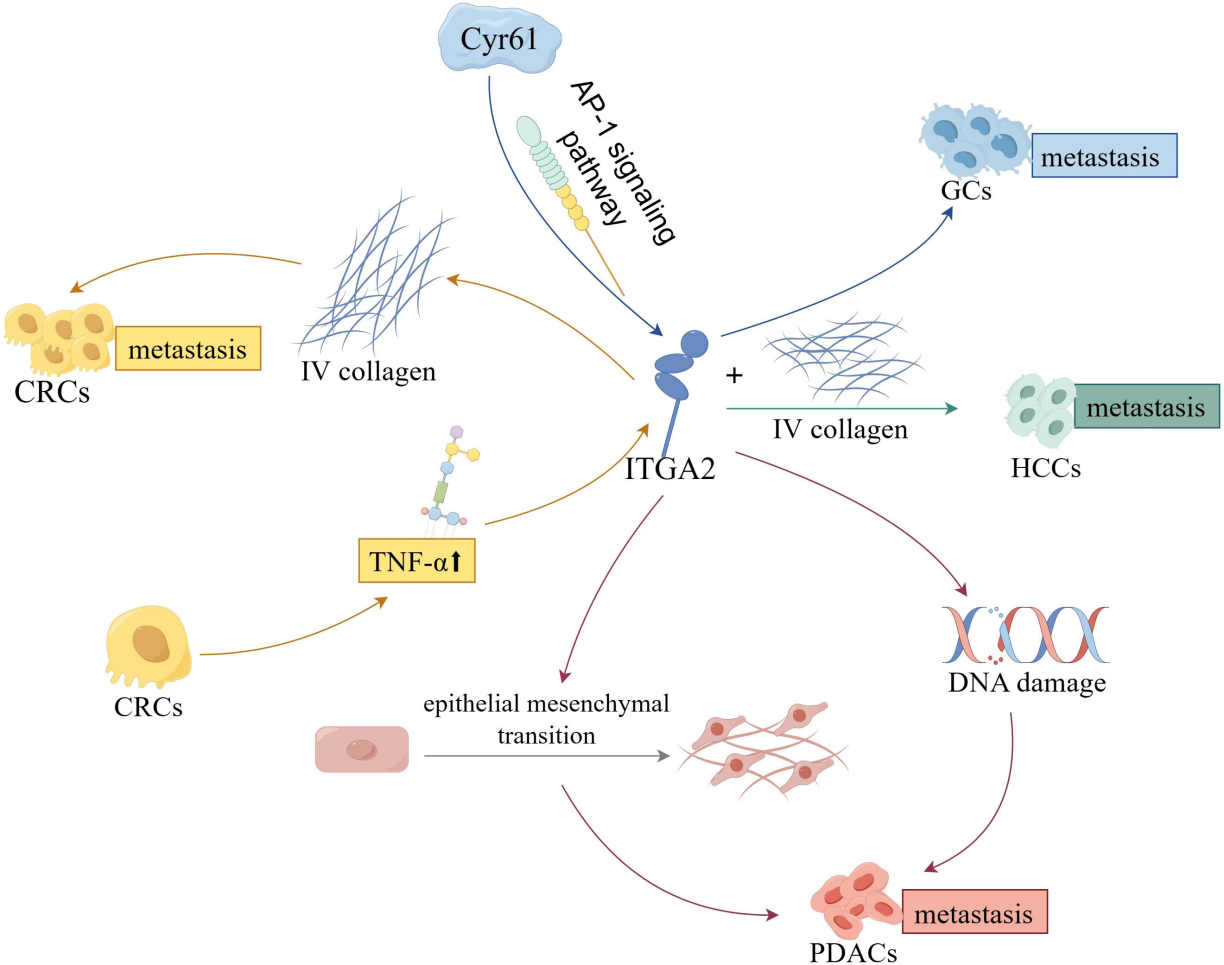
The effect and mechanisms of ITGA2 on digestive cancers.

In the context of digestive cancers, the primary role of ITGA2 lies in the regulation of tumor cell’s malignant biological behavior, subsequently influencing the progression, metastasis, and resistance to chemotherapy (**Figure 3**). Recently, there has been a growing body of research focusing on investigating the biological significance of ITGA2 within the TME.

Within the TME, regulatory T cells (Tregs) exert immunosuppressive effects through diverse mechanisms, such as the secretion of immunosuppressive mediators like IL-10, TGF-β, and adenosine, as well as the consumption of IL-2, which leads to the down-regulation of co-stimulatory molecules. ITGA2 has been identified as a marker indicating the functional maturity of Tregs. However, the elimination of ITGA2 in Tregs did not reveal any redundant role of ITGA2 itself in the function or migration of Tregs (41). Type 1 regulatory T (Tr1) cells exhibit heightened expression of the immunosuppressive cytokine IL-10 (42), thereby exerting inhibitory effects on inflammation and fostering immune tolerance. Additionally, ITGA2 serves as a molecular marker of Tr1 cells. Previous studies have provided evidence indicating that Tr1 cells and Tregs, which are capable of producing IL-10, exhibit the ability to co-express LAG3 and ITGA2 following differentiation (43–45). The studies listed above suggest that ITGA2 is commonly recognized as a surface marker in suppressive T cells and does not possess a distinct biological role.

CD4+T cells are the foundation for enduring immunity and efficacious secondary responses following infection or vaccination. CD4+T cells harboring antigen-specific memory predominantly reside within the bone marrow, with the presence of ITGA2 being imperative for the migration and survival of memory CD4+T precursor cells in this location (46). Recent investigations have revealed that tumor-infiltrating CD8+T cells initially exhibit ITGA2 expression, which subsequently facilitates the repositioning of T cells within the TME and induces CD8+T cell dysfunction (47). In the TME, ITGA2 primarily governs the motility of infiltrated T cells, potentially through its interaction with collagen. ITGA2 has been identified as a potential marker for NK cells. However, it is not deemed essential for the differentiation and effector function of NK cells (48). Nonetheless, an *in vitro* experiment has demonstrated that ITGA2 can impede the motility of NK cells, thereby restricting their ability to remodel and lyse target cells *in vitro (* 49). In the context of TME, the up-regulation of immune checkpoints plays a pivotal role in facilitating cancer immune evasion (50). Recent research findings indicate that targeting ITGA2 leads to an augmentation in the population of tumor-killing lymphocytes, while concurrently reducing the proportion of immunosuppressive cells within the tumor. This phenomenon is attributed to the ability of ITGA2 to enhance the phosphorylation of STAT3 in cancer cells, thereby initiating the expression of PD-L1 (51). Consequently, ITGA2 emerges as a promising candidate for enhancing the effectiveness of immunotherapy. Further investigation is warranted to ascertain ITGA2’s potential as a novel immunotherapeutic target for malignant tumors. Considering its role as a facilitator of cancer progression in gastrointestinal tumors, it is plausible that ITGA2 hampers the immune cells’ antitumor capabilities within the TME by modulating their mobility and immune checkpoints.

Cancer-associated fibroblasts (CAFs) are central cell types in the TME by exerting control over tumor growth, angiogenesis, metastasis, and immunosuppression (52). Similar to T cells and NK cells, ITGA2 can serve as a surface marker for identifying CAFs (53). CAFs are major producers of paracrine signals in TME. CAFs secrete an interstitial glycoprotein called TSP-4, which binds to ITGA2 receptors on tumor cells. The binding event results in the phosphorylation of heat shock factor 1 at residue S326. This interaction exerts two-fold effects, namely the preservation of malignant traits in gallbladder cancer cells and the induction of TGF-β1 expression and paracrine signaling. Consequently, peritumoral fibroblasts undergo transformation into cancer-associated fibroblasts (CAFs), establishing a positive feedback loop that amplifies tumor malignancy (54). A single cell sequencing result based on HCC demonstrates that CAFs engage in interactions with tumor cells via the COL1A1-ITGA2 signaling pathway, leading to the activation of the YAP signaling pathway and the regulation of desmoplasia and transcriptional heterogeneity in cancer cells (36). The diverse mechanisms through which ITGA2 facilitates communication between CAFs and tumor cells highlight the importance of investigating the functional alterations of ITGA2 in the TME.

## 7 Discussion

As a major public health problem worldwide, cancer can threaten human health seriously. In addition to surgery, radiotherapy and chemotherapy, targeted therapy is also becoming progressively important in cancer treatment. Herein, it is of great necessity to explore the novel therapeutic targets and tumor markers.

ITGA2 acts as an important transmembrane receptor with bidirectional signal transduction properties. The abnormal activation of its signal is often closely related to common diseases such as cancer, thrombosis, chronic inflammation (55). In addition, studies have also revealed the potential role of integrin α2, α3 and β1 subunits in pancreatic ductal adenocarcinoma in cancer cell signaling. The collagen homotrimers-integrin α3 pathway also plays a crucial part in pancreatic ductal adenocarcinoma progression and inhibition of T cell infiltration (56). It has also been found that intestinal microbiota affects the migration of immunosuppressive T cells to tumors and the efficiency of tumor immunotherapy by regulating the interaction between mucosal addressin cell adhesionmolecule1 and the integrin α4β7 on immunosuppressive T cells (57). Therefore, the possibility that the microbiota may influence the prognosis of gastrointestinal tumors by regulating ITAG2 is worthy of further exploration.

Moreover, a number of studies have shown that the loss of ITGA2 can reduce the proliferation, invasion and adhesion of glioma cells, and can be targeted to overcome the resistance of glioma cells to radiotherapy and chemotherapy, thereby prolonging the survival of patients (10). This also makes ITGA2 an attractive target for the treatment of glioma. However, studies on ITGA2 in GC, pancreatic cancer, HCC, CRC and other gastrointestinal tumors are gradually increasing. Therefore, we mainly focus on the research of ITGA2 in gastrointestinal tumors and their microenvironment in this review, aiming to provide a potential new marker for guiding the treatment and prognosis of many gastrointestinal tumors.

However, the exact biological function and regulatory mechanism in the TME are still not supported, which needs more exact evidence. In the future, we believe that there will be more advanced science and technology as well as methods to comprehensively understand the effects of ITGA2 on the TME of different cell types. Moreover, there are also some researches suggesting that integrins have some side effects. For example, due to the complexity of the TME, the effect of ITGA2 may be interfered by other factors, making the drug development more difficult (55). It is also urgent to improve preclinical studies of ITGA2 in combination with other drugs. Also, finding new biomarkers for cancer progression and metastasis is a key point in cancer research nowadays.

## 8 Conclusions

In summary, ITGA2 plays a pivotal role in digestive cancers, exerting influence on cancer initiation, metastasis, resistance to chemotherapy, and genomic instability. Nevertheless, the precise biological function and regulatory mechanism of ITGA2 in the TME remain inadequately supported by robust evidence. In the future, employing advanced scientific methodologies, a comprehensive understanding of ITGA2 impact on diverse cell types within the TME can be elucidated. Consequently, targeting ITGA2 could potentially serve as a novel therapeutic approach to enhance therapies modulating/targeting the reprogram of the TME.
